# The Diabetic Heart: Too Sweet for Its Own Good?

**DOI:** 10.1155/2012/845698

**Published:** 2012-02-22

**Authors:** Hannah J. Whittington, Girish G. Babu, Mihaela M. Mocanu, Derek M. Yellon, Derek J. Hausenloy

**Affiliations:** The Hatter Cardiovascular Institute, University College London, 67 Chenies Mews, London, WC1E 6HX, UK

## Abstract

Diabetes mellitus is a major risk factor for ischemic heart disease (IHD). Patients with diabetes and IHD experience worse clinical outcomes, suggesting that the diabetic heart may be more susceptible to ischemia-reperfusion injury (IRI). In contrast, the animal data suggests that the diabetic heart may be either more, equally, or even less susceptible to IRI. The conflicting animal data may be due to the choice of diabetic and/or IRI animal model. Ischemic conditioning, a phenomenon in which the heart is protected against IRI by one or more brief nonlethal periods of ischemia and reperfusion, may provide a novel cardioprotective strategy for the diabetic heart. Whether the diabetic heart is amenable to ischemic conditioning remains to be determined using relevant animal models of IRI and/or diabetes. In this paper, we review the limitations of the current experimental models used to investigate IRI and cardioprotection in the diabetic heart.

## 1. Introduction

Ischemic heart disease (IHD) is the leading cause of death and disability in the UK. Diabetes mellitus is a major risk factor for IHD patients with diabetes who are 2-3 times more likely to develop IHD [1]. Diabetes mellitus (DM), in general, is a condition where the body cannot adequately control its level of glucose. There are two types of diabetes, type I diabetes and type II diabetes. Type I diabetes is characterized by the inability of the body to produce insulin; this is caused by cellular-mediated programming of the autoimmune system and subsequent destruction of pancreatic beta-cells, the cells responsible for insulin production. The prevalence of this form of diabetes is relatively low and usually starts early in life [2]. Whereas type II diabetes is known as the insulin resistant form, insulin is produced however the target tissues become insensitive or resistant to the action of insulin thereby not utilizing blood glucose correctly and as a consequence increasing circulating blood glucose levels [3]. This form accounts for a high percentage of all diabetes and is historically more common in older age groups; however the prevalence of type II diabetes in children and young adults is also increasing especially in westernized societies [4].

There are many features of the diabetic heart that can contribute to an increased susceptibility to IHD, these include diabetic cardiomyopathy and angiopathy [5]. Diabetic cardiomyopathy is a disease that affects the myocardium in diabetic patients, causing structural changes which lead to abnormal functionality of the heart. These changes can eventually result in left ventricular hypertrophy (LVH) and diastolic/systolic dysfunction [6]. Changes at the molecular level occur in the diabetic heart leading to this phenotype, including endothelial dysfunction, metabolic perturbations, and differences in cellular signaling, which are well described in an extensive review by Hayat et al., 2004 [6]. Diabetic angiopathy causes vascular complications in chronic diabetic patients and can be classified into two types: microangiopathy and macroangiopathy [7]. Diabetic microangiopathy is the term coined to describe the damage caused to small blood vessels and capillaries within the body as a consequence of chronic hyperglycemia. This damage can lead to a decreased supply of oxygen and vital substrates to the tissues, which can lead to adverse clinical outcomes such as retinopathy, nephropathy, neuropathy, and diabetic foot [8]. Macroangiopathy as a consequence of diabetes mainly involves an accelerated form of atherosclerosis [9], another risk factor for CV disease. It affects the larger blood vessels within the body, where development and progression of atherosclerotic plaques lead to stenosis or occlusions, impairing blood flow [10]. Diabetic complications, atherosclerosis, hypertension, and many other CV risk factors such as obesity can all interact and render the heart more susceptible to IHD [11].

 The incidence of diabetes is increasing at an alarming rate throughout the world. Globally, the estimated prevalence of diabetes for 2010 was 285 million and is expected to affect 438 million people by 2030 [1]. In the UK, there are 2.6 million people who have been diagnosed with diabetes (2009) which equates to a 4.1% average prevalence and it is estimated that 4 million people will be diabetic by 2025 [12].

The major consequence of IHD results from the detrimental effect of acute myocardial ischemia-reperfusion injury (IRI). IRI is a paradoxical event that occurs in the myocardium. Briefly, when blood flow is reduced due to myocardial ischemia, the best treatment strategy is to reestablish blood flow to the damaged area, however this return in blood flow causes damage [13]. Patients with diabetes experience worse clinical outcomes in a number of clinical settings of acute IRI including acute myocardial infarction (MI) [14–16], coronary angioplasty [16], and cardiac bypass surgery [17–19]. This clinical data suggests that the diabetic heart may be more susceptible to acute IRI. In contrast, the animal data is inconclusive with experimental studies suggesting that the diabetic heart may be more, equally or even less susceptible to acute IRI [20]. However, one major reason for the disparity between the clinical and animal data may be due to the choice of IRI and/or diabetic animal model used in the animal studies [20].

 Given the worse clinical outcomes in diabetic patients with IHD, novel therapeutic strategies for protecting the diabetic heart against the detrimental effects of acute IRI are required to improve clinical outcomes in this patient group. Extensive research has investigated protecting the heart from IRI using conditioning strategies, either by mechanically activating cell survival pathways by ischemic preconditioning (IPC) [21] and remote ischemic conditioning (RIC) [22] or pharmacologically using cardioprotective agents [23]. Ischemic conditioning is an endogenous phenomenon in which one or more brief cycles of nonlethal ischemia and reperfusion applied directly to the heart protects itself from a sustained lethal episode of acute IRI [24]. Furthermore exciting research in the area of RIC, whereby an alternative organ to the heart, for example, the arm, can be made ischemic by one or more brief cycles of nonlethal ischemia and reperfusion using a blood pressure cuff, can limit the damage caused by a sustained ischemic insult [22] and this may provide a novel cardioprotective approach for the diabetic heart. These strategies are further described in Section 4. However, in order to translate ischemic conditioning into the clinical arena for the benefit of diabetic patients, it is important to first determine in animal studies whether the diabetic heart is amenable to cardioprotection elicited by ischemic conditioning. To achieve this requires the use of appropriate animal models of IRI and diabetes which also take into account other comorbidities such as age, dyslipidemia, and hypertension, factors which also impact on the ability to ischemic condition the heart [25].

The aim of this paper will be to highlight the limitations of currently used animal models of IRI and diabetes as a potential explanation for the disparity that exists between clinical and experimental data, with regard to the susceptibility of the diabetic heart to IRI and endogenous cardioprotection.

## 2. Animal Models of Diabetes

Animal models used to investigate diabetes have been created using a variety of different methods such as the administration of drugs toxic to the pancreas, modified diets, inbreeding of spontaneous mutations, or genetic engineering [34]. This has resulted in the availability of many different diabetic animal models [35]. In addition, the diabetic status induced by these methods needs to be assessed with caution as none of the models accurately and completely reflect the human pathology of diabetes. 

For example, type I diabetes is characterized by insulin deficiency as a consequence of autoimmune destruction of pancreatic *β* cells in the islets of Langerhans [36]. This disease is mimicked by administration of streptozotocin (STZ). However, this is an alkylating agent based nitrosourea derivative [37], which interferes with numerous cellular processes such as glucose transport, glucokinase function and can also induce DNA strand breaks [38]. This toxic compound can be given either as a single high dose to induce diabetes but this is associated with high mortality. Therefore, it is more common to use a series of low doses to induce diabetes in rodents [39]. Alloxan is a toxic glucose analogue, which preferentially accumulates in beta cells of the pancreas, causing excessive production of hydroxyl free radicals and destruction of the beta-cells hence mimicking type I diabetes [40]. In essence, the primary etiopathology caused by these experimental approaches has little autoimmune component and does not truly reflect the pathogenesis leading to type I diabetes in the human [39]. Therefore, it could be argued that the translation of findings from this particular animal model of type I diabetes to the clinical setting may be problematic. 

Type II diabetes in humans is a metabolic disorder and normally arises during adulthood [41]. A good animal model of type II diabetes is the Goto-Kakizaki (GK) rat, which originates from the inbreeding of Wistar rats that exhibited hyperglycemia. The GK rat spontaneously becomes diabetic early in life, showing glucose insensitivity in their pancreatic beta cells [42] with the diabetic status increasing with age [43]. The Otsuka Long Evans Tokushima Fatty (OLETF) rat originates from the inbreeding of Long-Evans rats which exhibit glucose intolerance [44]; these rats are mildly obese and the diabetic phenotype is more dramatic in males. Both the Zucker diabetic fatty (ZDF) rat [45] and the *db/db* mice [46] are models that express other comorbidities such as obesity and dyslipidemia as well as glucose intolerance [47]. For a thorough review into animal models in diabetes please refer to [34]. 

## 3. The Susceptibility of the Diabetic Heart to Acute IRI

Despite the clinical data suggesting that the diabetic heart is more susceptible to acute IRI, the animal data has been conflicting with experimental studies showing more, equal, or less sensitivity to acute IRI. The reasons for this disparity between the animal and clinical data were the subject of a review in 1997 by Paulson, who concluded that the sensitivity of the diabetic heart to acute IRI was dependent on the animal models and conditions used. The diabetic heart was shown to be less sensitive to acute IRI in studies which (i) used a short duration of diabetes (<6 weeks); (ii) used glucose as the only substrate; (iii) used a no-flow IRI protocol, whereby global ischemia is initiated by total interruption of the perfusate to the heart. Whereas, if diabetes was more prolonged and severe, fatty acids were present in the perfusate and a low-flow IRI protocol was used that is, experimental protocols which reflect the clinical scenario better, the diabetic heart was found to be more sensitive to IRI [20]. We have reviewed the literature since 1997, and the same pattern emerges (see Tables 1, 2, and 3). However, to this we need to add another complicating factor, the choice of IRI model and the lack of other comorbidities such as age, dyslipidemia, and hypertension, factors which are critical when investigating cardioprotection. 

**Figure 1:**
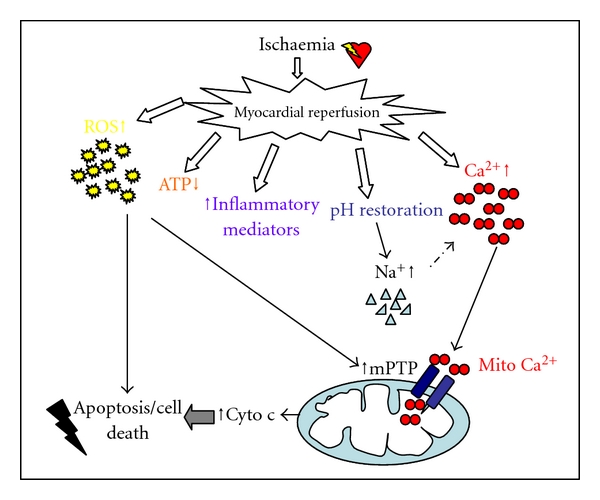
Endogenous factors contributing to ischemia-reperfusion injury. Following ischemia, blood flow is reestablished in the myocardium. The myocardium is subject to a number of abrupt changes during the transition from ischemia to reperfusion. Both biochemical and metabolic alterations occur including the generation of reactive oxygen species (ROS), decrease in ATP levels, an increase in inflammatory mediators, the rapid restoration of physiological pH, which in turn increases intracellular sodium and overload of intracellular calcium and mitochondrial calcium. These factors interact with each other to mediate reperfusion injury through the opening of the mitochondrial permeability transition pore (mPTP) and initiation of cell death pathways [13].

**Figure 2:**
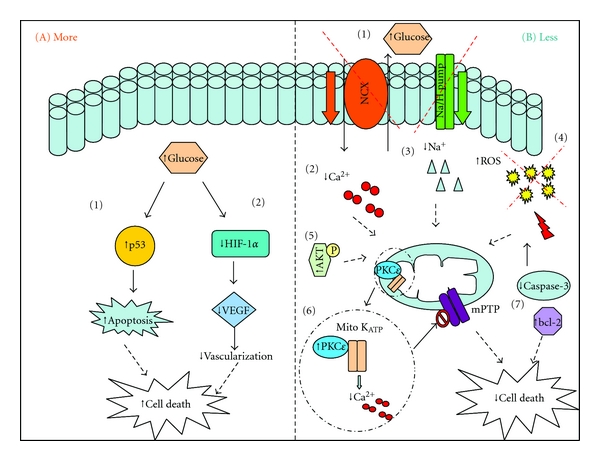
Possible mechanisms that make the diabetic heart more or less susceptible to infarction following ischemia reperfusion. (A) Diabetes can render the heart more susceptible to infarction. (A1) A diabetes-associated increase in the activity of p53, leading to the initiation of cell death pathway [29]. (A2) High-glucose causes a decrease in the activity of transcription factor HIF-1*α*, a subsequent downregulation of VEGF and less revascularization following ischemia [66]. This results in cell death and larger infarct volume. (B) Diabetes can protect the heart against infarction. (B1) Hyperglycaemia is cardioprotective due to the increased availability of glucose which is the hearts preferred substrate in times of stress. (B2/3) The Na^+^/Ca^2+^ and Na^+^/H^+^ exchangers in the diabetic heart reportedly have decreased activity; therefore the diabetic heart accumulates less of these ions preventing overload and the associated detrimental effects [20]. (B4) Diabetes is associated with an increased release of reactive oxygen species (ROS); a possible subsequent release of free radical scavenging enzymes increase the level of antioxidants within the myocardium protecting the heart from the consequence of IRI [20]. (B5) An increased basal level of prosurvival kinases in diabetes [57]. (B6) PKC-*ε* increases in diabetes, activating the mitochondrial K_ATP_ channel causing subsequent reduction in calcium accumulation and increasing ATP synthesis. PKC-*ε* also persistently translocate during ischemia but only in diabetic hearts [52]. (B7) High glucose caused reduction in cell death proteins and increased anti apoptotic bcl-2 [49].

Numerous endogenous factors contribute to myocardial IRI. These include production of reactive oxygen species (ROS), changes in the intracellular calcium and pH, triggering of inflammatory mechanisms; all of which interact with each other to mediate opening of the mitochondrial permeability transition pore (PTP), leading to eventual cardiomyocyte death (Figure 1) [13]. Interestingly, the pathological changes already occurring in diabetic cardiomyopathy include increased release of ROS [59], abnormal handling of calcium, and increased release of inflammatory mediators [60]; this experimental information alongside the evidence that diabetic patients have a worse prognosis following myocardial infarction [61], strongly suggest that the diabetic heart should be more vulnerable to damage. However, a plethora of experimental and clinical studies demonstrated a variety of outcomes, which could be highly dependent on the animal model, experimental protocol, and severity of diabetes. In Figure 2, we attempt to summarize the possible mechanisms that make the diabetic heart more or less susceptible to infarction following ischemia reperfusion. 

### 3.1. Animal Models of Type I Diabetes

A vast amount of research has been performed in order to assess the sensitivity to myocardial ischemic damage in models of type I diabetes. *In vivo *[53, 56] and *ex vivo *[58] investigations using a STZ-rat model demonstrated that acute diabetes (1–4 weeks of STZ treatment) resulted in less susceptibility to acute IRI. However, if STZ treatment was increased to longer than 6 weeks, the sensitivity to acute IRI was increased. Ma et al. [56] found increased phosphorylation of the prosurvival kinase Akt and decreased levels of caspase-3, vascular endothelial growth factor (VEGF), and nitric oxide (NO) following 2 weeks STZ induction but the opposite finding following 6 weeks treatment, supporting the loss of the cardioprotective state after 6 weeks [56]. Nawata et al. (2002) [51] also found that 4 weeks of STZ-induced diabetes reduced sensitivity to acute IRI in the Langendorff-perfused isolated heart model [51]. This phenomenon of protection in the acute diabetic setting was also present in alloxan-induced diabetes in Yucatan pigs [57]. Following 1 hour of regional coronary artery occlusion and reperfusion the *in vivo* myocardial infarct size was smaller compared to control and this was accompanied by an increased expression of cell survival proteins. Of note, global left ventricular function was worse in diabetes; however function within the area at risk was better [57]. In a cardiomyocyte model of diabetes, cells were incubated for 3 days with either 5 mM or 25 mM glucose in the medium. High-glucose treatment was protective against simulated IRI. The high-glucose treatment caused a reduction in necrosis, apoptosis, and calcium content, whereas the antiapoptotic protein bcl-2 increased and proapoptotic bad was shifted to its inactive state [49] in the presence of 25 mM glucose. 

In contrast to this data, showing that “acute” or short-term induced type I diabetes is cardioprotective compared to the longer-term induction, Hadour et al. (1998) [48] induced diabetes in rabbits for 8 weeks using alloxan, then subjected the hearts to *in vivo *30 minute myocardial ischemia and 3 hours reperfusion and saw a reduction in MI size compared to control nondiabetic rabbits. Conversely, in nondiabetic rabbits infused with high glucose throughout myocardial ischemia and reperfusion to mimic acute diabetes no difference in infarction was seen compared to controls. They suggested that the presence of type I diabetes in the rabbit induced a chronic and metabolic cardioprotective state in the heart [48]. Ebel et al. [31] showed in the same model of alloxan-induced diabetes in rabbits that 6-weeks duration of diabetes had no influence on the vulnerability to IRI compared to controls, further supporting the theory of a chronic (greater than 6 weeks) protected state in this model [31]. Chronic administration of STZ for 12 weeks in rats also showed a decreased susceptibility to MI [52]. Diabetic hearts had greater left ventricular function and the incidence of ventricular fibrillation and creatine kinase (CK) release were decreased. This was accompanied by a persistent translocation of protein kinase C-*ε* (PKC-*ε*), a modulator of the mitochondrial permeability transition pore, during ischemia but only in the diabetic hearts. Following these results, the authors suggested that in STZ-induced diabetes PKC-*ε* plays a crucial role in the susceptibility to MI [52]. However, this investigation was performed using a Langendorff-perfused model of no flow ischemia and contained no added substrates. It would be interesting to assess translocation properties of PKC-epsilon in the *in vivo* setting. 

Other investigators have also shown that acute initiation of type I diabetes does not influence the susceptibility to MI. Both alloxan and STZ-treated dogs, although at a lower dose to previously mentioned investigations, reported no difference in MI size following IRI *in vivo *[62]. 

However, in other studies, type I diabetes has been shown to render the heart more susceptible to IRI. It has been suggested that hyperglycemia has a negative impact on endogenous cardioprotective signaling. Kersten et al. [27] showed that hyperglycemia in dogs, induced either acutely by 15% dextrose prior to IRI *in vivo, *or in chemically induced diabetes (3 weeks STZ), increased MI size. Interestingly, the studies mentioned above suggested that “acute” diabetes would render the heart less susceptible to diabetes. However, those studies were in rodent models of diabetes and had a shorter duration of IRI. Ebel et al. [31] recorded a greater susceptibility to MI in their rabbit model following an infused solution of 50% dextrose 30 minutes prior to ischemia *in vivo* to elicit a hyperglycemic state of 600 mg/dL [31]. Su et al. [32] used an infused glucose model to elicit hyperglycemia in rats, this also rendered the heart more susceptible to MI of note the reperfusion time following ischemia in this study was notably longer compared to other rodent models [32]. With regard to the possible mechanism, hyperglycemia in rat cardiomyocytes was shown to promote p53-dependent activation of apoptosis [29]. 

Marfella et al. [30] also examined the effect of hyperglycemia and STZ-induced diabetes on MI size in rats, both *in vivo* and *ex vivo*. Both hyperglycemic conditions caused an increase in MI size, together with a decreased transactivation of the hypoxic inducible factor HIF-1*α* [30]. The diabetic induction in this model was 9 days prior to the ischemic episode, although an increased dose of STZ (70 mg/kg) was used. HIF-1*α* controls the upregulation of vascular endothelial growth factor (VEGF) in response to hypoxia, initiating neovascularization following ischemia. If this axis is impaired, as seems the case in diabetes, inadequate revascularization will occur resulting is potential worse outcomes for the diabetic patient [66].

### 3.2. Animal Models of Type II Diabetes

In 2004, Kristiansen et al. [54] were the first to investigate the susceptibility to acute IRI and ischemic conditioning (IPC) in a model of type II diabetes. Two distinct models: the GK rat and ZDF rat showed in Langendorff-perfused isolated hearts (with no added substrates), that MI size was smaller than their respective nondiabetic controls. Interestingly, even though these hearts appeared to be more resistant to ischemic damage, they were not amenable to protection by IPC [54]. Desrois et al. performed two separate studies also using the *ex vivo* Langendorff preparation in the GK rat in 2004 [63] and 2010 [33]. In 2004, they compared gender difference within diabetes and the effect this has on the susceptibility to MI. The preparation involved perfusing the hearts with Krebs-Hensleit buffer with no added substrates. They found that female hearts had larger MI size than the male GKs and that no significant difference was noted between male hearts of the diabetic and nondiabetic controls [63]. Interestingly, their study in 2010 again utilized the Langendorff technique, and showed that the male GK heart was more susceptible to MI than the control heart [33]. The latter investigation included additional fatty acid substrates in the perfusate, similar to those likely to be found in the *in vivo* scenario. This may suggest that in the setting of type II diabetes, substrates found in the blood could play an important role in cell damage. Studies in *db/db* mice *in vivo* support this idea; Lefer et al. (2001) [28] and Jones et al. (1999) [26] both saw a diabetes-associated increase in MI size. 

Other studies show a similar result to Kristiansen et al's study in 2004 [54]. Tsang et al. (2005) using the isolated heart model, with no added substrates, however with a shorter ischemic time, showed the heart of the GK rat to be less susceptible to infarction than the Wistar rat control [55]. Supporting these findings, cardiomyocytes isolated from GK rats were less susceptible to mPTP opening in response to calcium, achieved by adding soluble Ca^2+^ to a phosphate-containing medium. This was accompanied by a larger calcium accumulation, leading to decreased opening of the mitochondrial pore and reduced cardiomyocyte death [50]. Controversially, two recent investigations that have been performed *in vivo *[64, 65] in the GK rat showed no difference in the susceptibility to infarct compared to control rats following 30 or 35 minutes ischemia and 2-hour reperfusion. 

Some blood components, such as platelets and neutrophils, have been suggested to play a role as mediators of cell damage in ischemia and reperfusion [20]. This is another confounding factor when comparing data from cell, *in vitro* and *in vivo* experiments. The sensitivity of the type II diabetic heart in *in vivo* settings has had limited study compared to type I diabetes, therefore more work is required to clarify these initial findings.

## 4. Cardioprotection in the Diabetic Heart

### 4.1. Animal Models of IRI

The heart, as in any living tissue, has endogenous protective mechanisms which, when activated, render it resistant to IRI, in other words the heart can be “conditioned” [21]. Initial studies by Murry et al. (1986) [24], showed that short bursts of ischemia followed by reperfusion prior to a prolonged insult of ischemia and reperfusion, were associated with a reduction in MI size by 75%. This was termed “ischemic preconditioning” (IPC) [24]. The phenomenon of IPC is highly reproducible in all species [21]. Over the years, the phenomenon of IPC has evolved to include pharmacological conditioning [68], which involves targeting cellular mechanisms involved in IRI or promoting those involved in IPC. Also, short bursts of ischemia and reperfusion following an ischemic insult can reduce MI size and this term was coined ischemic postconditioning (IPost) [69]. More recently, remote ischemic conditioning has been discovered, whereby an organ or tissue remote from the heart is conditioned and reduces infarct size [22]. 

**Figure 3:**
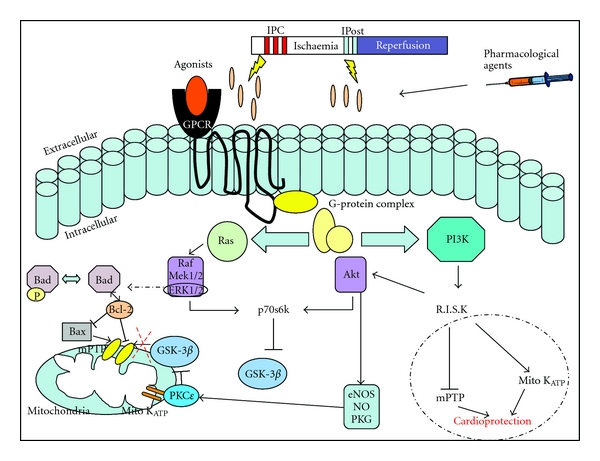
The cellular mechanisms involved in Ischemic Preconditioning. IPC, IPost, or pharmacological agents initiates the release of G-protein-coupled receptor (GPCR) agonists which bind to the receptor and activate numerous signaling pathways. Phosphatidylinositol-3-kinase (PI3K) and Ras activation can lead to activation of a number of downstream molecules such as Akt, protein kinase C (PKC), extracellular regulated kinase (ERK), nitric oxide synthase (NOS), and inactivation of glycogen synthase kinase-3*β* (GSK-3*β*). These converge to activate the mitochondrial ATP-dependent potassium channel (K_ATP_), closing the mitochondrial permeable transition pore (mPTP) resulting in protection from IRI [67].

IPC reduces lethal cell injury in the ischemic myocardium [24]; how this phenomenon works has been extensively studied and is summarized in Figure 3. Briefly, IPC causes the release of G-protein-coupled receptor (GPCR) agonists which bind to the receptor and activate numerous signaling pathways. Phosphatidylinositol-3-kinase (PI3K) activation can lead to activation of a number of downstream molecules such as Akt, protein kinase C (PKC), extracellular regulated kinase (ERK), nitric oxide synthase (NOS), and inactivation of glycogen synthase kinase-3*β* (GSK-3*β*). These converge to activate the mitochondrial ATP-dependent potassium channel (K_ATP_), closing the mitochondrial permeable transition pore (mPTP) resulting in protection from IRI [67]. Before IPC can be applied in the clinical setting it is important to determine whether diabetic heart is amenable to this endogenous cardioprotective strategy. The animal data suggests that the diabetic heart is resistant to ischemic conditioning such that the IPC stimulus needs to be increased to induce cardioprotection [70]. 

Bouchard et al. (1998) [70] showed that in a STZ rat model of diabetes, an IPC stimulus consisting of 1 cycle of 5-minute ischemia followed by 10-minute reperfusion (IPC-1) was not sufficient to preserve the vasodilatory response to 5-HT suggesting an impairment of endothelial cells in the diabetic heart and consequently the inability to generate and release enough NO. However, when the IPC stimulus was increased to 3 cycles of 5-minute ischemia followed by 5-minute reperfusion (IPC-3) the vasodilatory response to 5-HT was present. Furthermore, in the same investigation, 30-minute pretreatment with adenosine, an important mediator in IPC [71], mimicked the cardioprotective outcome of IPC-3, whereas 15-minute pretreatment was ineffective [70]. These findings suggested that signaling pathways activated by adenosine either endogenously or exogenously may be hindered in diabetic coronary vessels, but have the potential to be recovered by an increased stimuli. The K_ATP_ channel is involved in the mechanism of protection by IPC [72] and adenosine [73]; studies in diabetic animals have reported reduced sensitivity to K_ATP_ channel activators [74] which could correlate with the need for an increased stimulus to achieve K_ATP_ channel activation, subsequent mPTP inhibition, and reduction in apoptosis initiation hence reducing myocardial cell death [75]. Another study using alloxan-/STZ-induced diabetes in dogs also supported the role of dysfunctional K_ATP_ channel in diabetes [76]. 

Other important endogenous mechanisms involved in protection by IPC is the reperfusion injury salvage kinase pathway (RISK) [77], with its main constituent the PI3K-Akt axis. Tsang et al. (2005) [55] found that to protect the myocardium in the Goto-Kakizaki rat model of type II diabetes, an increased IPC stimulus was also required. Similar to the previous study, 1 cycle of 5-minute ischemia followed by 10-minute reperfusion was insufficient to protect the heart; however 3 cycles of this protocol reduced infarct volume considerably. They investigated the level of phosphorylated Akt (Akt-P) within the myocardium and found that in the diabetic heart, an increased IPC stimulus is required to achieve an essential level of Akt phosphorylation necessary to mediate cardioprotection [55], thereby highlighting a diabetes-associated impairment in PI3K-Akt signaling. Increasing the stimulus of IPC by increasing cycle number appears important in diabetes; however, the duration of the pre-ischemia/reperfusion protocol may also play a vital role. Four cycles of 2-minute ischemia followed by 3-minute reperfusion did not elicit protection in two separate models of diabetes [54], compared to 3 cycles of 5-minute ischemia and 10-minute reperfusion used by Tsang et al. [55] in the same animal and experimental setting. 

A recent study by Hotta et al. (2010) [78] using the OLETF rat model of type II diabetes showed that pretreatment with either opioid agonist [79] or erythropoietin (EPO) [80], known to mediate IPC signaling through Janus kinase (Jak-2), failed to elicit cardioprotection *in vivo* due to insufficient phosphorylation of Jak-2 and Akt, and like others, also found an elevated level of calcineurin activity in diabetic hearts [81]. Interestingly, following 2-week treatment with either valsartan or losartan, Angiotensin II receptor type 1 (AT1) receptor blockers, Jak-PI3K-Akt signaling, and hence cardioprotection subsequent to EPO administration were restored [78]. Huisamen et al. (2011) supported the finding that AT1 antagonism can lead to cardioprotection in their *ex vivo* rat model of insulin resistance, diet-induced obesity (DIO) rat [82]. 

In contrast, Ghaboura et al. (2011), examined EPO-induced IPost cardioprotection in two rat models of type I diabetes; STZ-induced and high-fat-diet (HFD-) induced insulin resistance syndrome. They found that EPO administered at the onset of reperfusion did not increase phosphorylation of Akt, ERK, or GSK-3*β* and hence did not elicit a cardioprotective effect in the hearts isolated from STZ rats; interestingly, HFD rat hearts were protected following EPO. Administration of a GSK-3*β* antagonist, a kinase which is downstream of the PI3K-Akt axis, given prior to ischemia and continued throughout reperfusion had an infarct limiting effect [83]. In support of the idea that inhibiting GSK-3*β* in diabetic hearts as a direct cardioprotective strategy, Gross et al. (2007) [84] showed that diabetes had a detrimental effect on morphine-induced protection. STZ-induced diabetes caused alterations in pathways upstream of GSK-3*β*, such as PI3K, Jak/STAT, and MAPK pathways, limiting the cardioprotective effects of morphine administered at reperfusion. Similarly, downstream antagonism of GSK-3*β* was cardioprotective [84]. The direct inhibition of GSK-3*β* was also proved cardioprotective in a rat model of type II diabetes [85]. In this study, another diabetic-associated myocardial phenotype was reported; they found that increased endoplasmic reticulum stress, caused alterations in PI3K-Akt, ERK, and GSK-3*β* signaling motifs, and hence dysregulation of the mPTP leading to the absence of cardioprotection by EPO [85]. 

The idea that the diabetic heart is still amenable to protection but has an increased threshold for the necessary activation of pro survival kinases is an interesting prospect. Investigations by Tsang et al. [55] and Bouchard et al. [70] clearly demonstrated this; however, in the study by Ghaboura et al. [83] increasing the dose of EPO did not restore cardioprotective effects. Of course, these studies are performed in a variety of models of diabetes, with variable levels of diabetic severity. Tsang et al. [55] used a model with moderate hyperglycemia, however both Bouchard et al. [70] and Ghaboura et al. [83] induced diabetes by injections of STZ at 55 mg/kg and 65 mg/kg, respectively. Therefore, speculation that the ability to protect the diabetic heart can be restored by an increased stimulus in moderate hyperglycemia but not severe hyperglycemia could be flawed and needs further investigation. 

All of the mentioned studies have furthered the understanding of the mechanisms that could render the diabetic heart ineffective to cardioprotective strategies, and how we could potentially overcome these hurdles. Though a variety of animal models were used, including *in vivo* and *ex vivo *experimental protocols, direct targeting of GSK-3*β* in the diabetic heart may provide an alternative solution to prevent ischemia reperfusion injury [86]. However, like any therapy, a targeted approach would have to be considered to limit any potential negative effects [87].

### 4.2. Experimental Studies Using Human Heart Tissue

As part of the translational process, our laboratory have established since 1995 an isolated human atrial muscle preparation of simulated IRI [90]. This preparation has become a popular tool in investigating the response of human tissue in cardiovascular diseases. In brief, samples of right atrial appendage are obtained from the right atrial cannula insertion site in patients undergoing cardiac bypass during coronary artery bypass graft surgery [91]. From the right atrial appendage, trabeculae are isolated and subjected to simulated IRI and the recovery of basal function assessed following IRI. 

Numerous studies have been performed investigating the mechanisms of preconditioning in the normoglycemic human myocardium [92]; however a limited number of studies also investigated this phenomena in the diabetic human heart. Using a variation of this model in which the right atrial appendage is sliced and subjected to simulated IRI, Ghosh et al. (2001) [93], showed the failure of IPC to protect the diabetic human myocardium with 1 cycle of 5-minute ischemia and 5-minute reperfusion reflecting what was already demonstrated in animal investigations, that is, an increased threshold for cardioprotection in the hyperglycemic tissue. As previously mentioned K_ATP_ channels play a role in IPC. Diazoxide, a K_ATP_ opener, did not mimic IPC in diabetic tissues suggesting a dysfunctional mitochondrial K_ATP_ channel [93]. This result was supported by the finding that the ATP-sensitive potassium channels are altered in ventricular myocytes from diabetic rats [94]. Of interest, this group did not try increasing the IPC stimulus to see if the human muscle mirrored the finding from Tsang and colleagues [55]. Hassouna et al. (2006) also suggested that the dysfunctional mitochondrial K_ATP_ channel exists in diabetics, causing impaired depolarization and superoxide production, resulting in an inability to respond to IPC [95]. Increasing the IPC stimulus to 3 cycles in this experimental model did not restore the protection [95], conflicting with the finding of Tsang et al. [55]. However, Sivaraman et al. (2010) [88] showed that increasing the duration of ischemic period of IPC from 4 minutes to 7 minutes followed by reoxygenation in human diabetic atrial tissue restored cardioprotection and similarly was related to a downregulation of PI3K-Akt axis in diabetic tissue [88]. 

In a recent study performed by Linares-Palomino et al. specific inhibitors of Akt were utilized to delineate its specific role in ischemic pre conditioning. They showed that blockade of Akt caused a significant reduction in cell death, similar to the degree of protection elicited by either IPC or PI3K inhibition and this was evident in both rat and human tissue. Interestingly, they showed Akt to be downstream of mitochondrial K_ATP_ channel but upstream of p38 MAPK [96]. 

**Figure 4:**
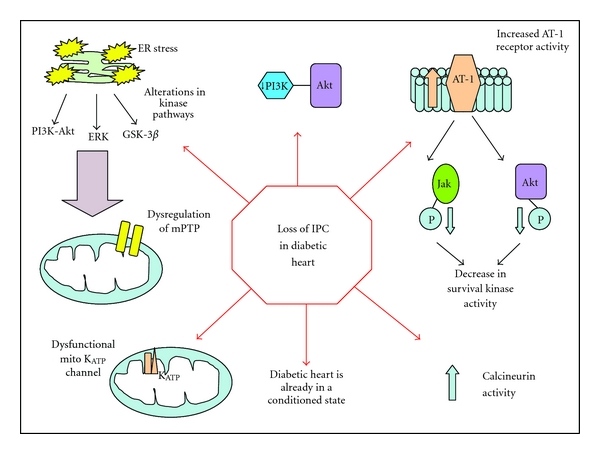
Why is the diabetic heart harder to protect with conditioning strategies? The diabetic heart has been suggested to have a raised threshold for cardioprotection [55], this is caused by the downregulation of prosurvival kinase pathways [55, 88], resulting in dysregulation of mitochondrial permeability transition pore (mPTP), increased receptor activities for pharmacological agents [78], increased calcineurin activity [81] and evidence suggests a dysfunctional K_ATP_ channel in the mitochondria [76]. In diabetes, endoplasmic reticulum (ER) stress also causes alterations in kinase pathways leading to dysregulation of the mPTP [85]. Interestingly, some evidence suggests that the diabetic heart is in a paradoxical protective state therefore conditioning potential is lower [89].

 This data reinforces the fact that cardioprotection in the diabetic heart is a complex and delicate phenomenon; the knowledge gained from the extensive research throughout many models has highlighted some potential reasons why it is more difficult to protect the diabetic heart, reasons which are summarized in Figure 4. 

 Proof-of-concept clinical studies are now required to determine whether the human diabetic heart is amenable to cardioprotection elicited by ischemic conditioning. Preliminary unpublished data from our research group suggests that the diabetic patient may not be amenable to cardioprotection elicited by remote ischemic conditioning (RIC). There was no difference in perioperative myocardial IRI (72 hour area under the curve serum Troponin T) in diabetic patients randomized to receive a standard RIC stimulus (three 5-minute inflations and deflations of a blood pressure cuff on the upper arm) prior to CABG surgery (Babu et al. unpublished), a RIC protocol which has been previously reported to protect nondiabetic patients [97].

## 5. Limitation of Translation and Future Directions

The obvious limitation of translating research from bench to bedside is that many of the animal models we use do not adequately reproduce the clinical setting or fully represent the disease pathology and manifestation in patients. In addition, the vast majority of studies are conducted on inbred animals with very little genetic variability; they are fed ad libitum with scientific diets and are kept in optimal living conditions of light and temperature. This is far from the complexity of patients who possess a large genetic pool, undergo the stresses of day to day working life, some eating high-fat diets and undertaking limited exercise. In addition, the majority of people in westernized societies take a plethora of medications. 

 There is an increasing amount of research now being conducted in animals with comorbidities to promote successful translation of the research field of cardioprotection. The design of the preclinical model, either in human tissue or animal models, is critical to harness the huge potential of IPC as a strategy to reduce ischemic damage. Preliminary unpublished data from our research group, utilizing aging, diabetic rats suggests an age-associated increase to the threshold of IPC and susceptibility to infarction in models of type II diabetes (Whittington et al. unpublished). These results could highlight a possible discrepancy from original IPC studies performed in young healthy animals and could indicate why the findings were not transferable to the clinic.

## 6. Summary

It is well established that diabetic patients with IHD experience worse clinical outcomes yet the animal data is conflicting. The choice of the diabetic and IRI animal model may in part explain this discrepancy. The development of novel cardioprotective strategies for protecting the diabetic heart and improving clinical outcomes in diabetic patients with IHD will be dependent on using relevant animal models of IRI and diabetes which also take into account other comorbidities such as age, dyslipidaemia, and hypertension. Therefore, by carefully selecting and optimizing clinically relevant animal models of IRI and diabetes, we may be able to better translate findings made at the “bench” to patients' “bedside”.

## Figures and Tables

**Table 1:** Studies indicating the diabetic heart is more sensitive to ischemic injury compared to normoglycemic controls.

Study	Model	Ischemic protocol	Duration/onset of diabetes	Substrates	Model of diabetes	End points
Jones et al. (1999) [26]	*db/db* mouse	*In vivo* non recovery, 30 min regional ischemia/2 h reperfusion	In-bred strain	*In vivo* substrates	Type II diabetes	Infarction

Kersten et al. (2000) [27]	Dog, Alloxan (40 mg/kg) and STZ (25 mg/kg)	*In vivo* non recovery, 60 min regional ischemia/3 h reperfusion	3 weeks	*In vivo* substrates	Type I diabetes	Infarction

Kersten et al. (2000) [27]	Dog, Dextrose 15% to cause acute hyperglycaemia	*In vivo* non recovery, 60 min regional ischemia/3 h reperfusion	70 mins	*In vivo* substrates	Type I diabetes	Infarction

Lefer et al. (2001) [28]	*db/db* mouse	*In vivo* non recovery, 30 min regional ischemia/2 h reperfusion	In-bred strain	*In vivo* substrates	Type II diabetes	Infarction

Fiordaliso et al. (2001) [29]	Rat cardiomyocytes	—	1, 2, and 4 days of 25 mmol/L incubation in medium	—	Type I diabetes	Cell death

Marfella et al. (2002) [30]	Sprague-Dawley Rat, STZ (70 mg/kg i.v)	*In vivo* non recovery, 25 min regional ischemia/2 h reperfusion	9 days	*In vivo* substrates	Type I diabetes	Infarction and protein expression

Marfella et al. (2002) [30]	Sprague-Dawley Rat, isolated heart	Langendorff isolated heart, 25 min regional ischemia/2 hr reperfusion	—	33.3 mmol/L glucose	Type I diabetes	Infarction and protein expression

Ebel et al. (2003) [31]	Rabbit- 50% Dextrose infused 30 min prior to ischemia until reperfusion normoglycaemic rat-under intravenous	*In vivo* non recovery, 30 min regional ischemia/2 h reperfusion	hyperglycemia of 600 mgd1-1 throughout ischemia	*In vivo* substrates	Type I diabetes	Infarction
Su et al. (2007) [32]	infusion at a rate of 4 mL*·*kg–1*·*h–1: of glucose 500 g/l during ischemia, saline during reperfusion	*In vivo* non recovery, 30 min regional ischemia/6 h reperfusion	—	*In vivo* substrates	Type I diabetes	Infarction, apoptosis and kinase expression

Desrois et al. (2010) [33]	Aging Goto Kakizaki Rat, male	Langendorff isolated heart, 32 min low flow global ischemia/32 min reperfusion	In-bred strain	1.2 mM palmitate, 3% albumin, 11 mM glucose, 3 U/l insulin, 0.8 mM lactate, and 0.2 mM pyruvate.	Type II diabetes	Myocardial function

**Table 2:** Studies indicating the diabetic heart is less sensitive to ischemic injury compared to normoglycaemic controls.

Study	Model	Ischemic Protocol	Duration/onset of diabetes	Substrates	Model of diabetes	End points
Hadour et al. (1998) [48]	Rabbit, alloxan (100 mg/kg)	*In vivo* nonrecovery, 30 min regional ischemia/3 hr reperfusion	2 months	*In vivo* substrates	Type I diabetes	Infarction

Schaffer et al. (2000) [49]	Rat, neonatal cardiomyocytes	10 mM deoxyglucose and and 3 mM amobarbital medium for 1 hr, OR hypoxic chamber: 2.3% O2–5% CO2-balance N2 for 1 hr	3-day incubation with 25 mM glucose in medium	—	Type I diabetes	Infarction

Oliveria et al. (2001) [50]	Goto Kakizaki Rat, male, isolated cardiomyocyte mitochondria	—	In-bred strain	—	Type II diabetes	Cell death and mPTP

Nawata et al. (2002) [51]	Rat, STZ (65 mg/kg)	Langendorff isolated heart, 30 min low flow global ischemia/30 min reperfusion	4 weeks	11 mmol/L glucose	Type I diabetes	Myocardial function

Ooie et al. (2003) [52]	Rat, STZ (65 mg/kg)	Langendorff isolated heart: Low-flow global ischemia for 5 min, followed by no-flow ischemia for 25 min. 30 min reperfusion	12 weeks	11 mmol/L glucose	Type I diabetes	Myocardial function, creatine kinase release

Ravingerová et al. (2003) [53]	Rat, STZ (45 mg/kg)	*In vivo* non recovery, 30 min regional ischemia/4 hr reperfusion	1 week	*In vivo* substrates	Type I diabetes	Infarction

Kristiansen et al. (2004) [54]	Goto Kakizaki Rat, male	Langendorff isolated heart, 50 min regional ischemia/2 hr reperfusion	In-bred strain	11 mmol/L glucose	Type II diabetes	Infarction

Kristiansen et al. (2004) [54]	Obese Zucker Diabetic Fatty Rat, male	Langendorff isolated heart, 50 min regional ischemia/2 hr reperfusion	In-bred strain	11 mmol/L glucose	Type II diabetes	Infarction

Tsang et al. (2005) [55]	Goto Kakizaki Rat, male	Langendorff isolated heart, 30 min regional ischemia/2 hr reperfusion	In-bred strain	11 mmol/L glucose	Type II diabetes	Infarction, kinase expression

Ma et al. (2006) [56]	Rat, STZ (50 mg/kg)	*In vivo* non recovery, 30 min regional ischemia/2 hr reperfusion	2 weeks	*In vivo* substrates	Type I diabetes	Infarction

Chu et al. (2010) [57]	Yucatan pigs, alloxan (200 mg/kg)	*In vivo* non recovery, 1 hr regional ischemia/2 hr reperfusion	5 weeks	*In vivo* substrates	Type I diabetes	Infarction and protein expression

Shi-Ting et al. (2010) [58]	Rat, STZ (60 mg/kg)	Langendorff isolated heart, 30 min regional ischemia/40 min reperfusion	4 weeks	11 mmol/L glucose	Type I diabetes	Infarction and creatine kinase release

**Table 3:** Studies indicating no difference in the sensitivity of the diabetic heart to ischemic injury compared to normoglycemic controls.

Study	Model	Ischemic Protocol	Duration/onset of diabetes	Substrates	Model of diabetes	End points
Hadour et al. (1998) [48]	Rabbit, 10% glucose infusion to 300 mg/dL blood glucose	*In vivo* nonrecovery, 30 min regional ischemia/3 hr reperfusion	Blood glucose maintained at 300 mg/dL throughout procedure	*In vivo* substrates	Type I diabetes	Infarction

Tanaka et al. (2002) [62]	Dog, alloxan (40 mg/kg) and STZ (25 mg/kg)	*In vivo* nonrecovery, 60 min regional ischemia/3 hr reperfusion	3 weeks	*In vivo* substrates	Type I diabetes	Infarction

Ravingerová et al. (2003) [53]	Rat, STZ (45 mg/kg)	*In vivo* nonrecovery, 30 min regional ischemia/4 hr reperfusion	8 weeks	*In vivo* substrates	Type I diabetes	Infarction

Ebel et al. (2003) [31]	Rabbit- alloxan (100 mg/kg)	*In vivo* nonrecovery, 30 min regional ischemia/2 hr reperfusion	6 weeks	*In vivo* substrates	Type I diabetes	Infarction

Desrois et al. (2004) [63]	Aged Goto Kakisaki Rat, male	Langendorff isolated heart, 32 min low flow global ischemia/32 min reperfusion	In bred strain	11 mmol/L glucose	Type II diabetes	Myocardial function

Ma et al. (2006) [56]	Rat, STZ (50 mg/kg)	*In vivo* nonrecovery, 30 min regional ischemia/2 hr reperfusion	6 weeks	*In vivo* substrates	Type I diabetes	Infarction

Bulhak et al. (2009) [64]	Goto Kakizaki Rat, male	*In vivo* nonrecovery, 35 min regional ischemia/2 hr reperfusion	In bred strain	*In vivo* substrates	Type II diabetes	Infarction

Matsumoto et al. (2009) [65]	Goto Kakizaki Rat, male	*In vivo* nonrecovery, 30 min regional ischemia/2 hr reperfusion	In bred strain	*In vivo* substrates	Type II diabetes	Infarction

Shi-Ting et al. (2011) [58]	Rat, STZ (60 mg/kg)	Langendorff isolated heart, 30 min regional ischemia/40 min reperfusion	8 weeks	11 mmol/L glucose	Type I diabetes	Infarction and creatine kinase release

## References

[1] International Diabetes Federation (2009). *Diabetes Atlas*.

[2] Notkins AL, Lernmark A (2001). Autoimmune type 1 diabetes: resolved and unresolved issues. *Journal of Clinical Investigation*.

[3] Goldstein BJ (2002). Insulin resistance as the core defect in type 2 diabetes mellitus. *American Journal of Cardiology*.

[4] Fagot-Campagna A, Venkat Narayan KM, Imperatore G (2001). Type 2 diabetes in children: exemplifies the growing problem of chronic diseases. *British Medical Journal*.

[5] Butler R, Macdonald TM, Struthers AD, Morris AD (1998). The clinical implications of diabetic heart disease. *European Heart Journal*.

[6] Hayat SA, Patel B, Khattar RS, Malik RA (2004). Diabetic cardiomyopathy: mechanisms, diagnosis and treatment. *Clinical Science*.

[7] Schalkwijk CG, Stehouwer CDA (2005). Vascular complications in diabetes mellitus: the role of endothelial dysfunction. *Clinical Science*.

[8] Dandona P, Aljada A, Chaudhuri A, Bandyopadhyay A (2003). The potential influence of inflammation and insulin resistance on the pathogenesis and treatment of atherosclerosis-related complications in type 2 diabetes. *Journal of Clinical Endocrinology and Metabolism*.

[9] Colwell JA, Lopes-Virella M, Halushka PV (1981). Pathogenesis of atherosclerosis in diabetes mellitus. *Diabetes Care*.

[10] Ross R (1999). Atherosclerosis—an inflammatory disease. *The New England Journal of Medicine*.

[11] (1999). Diabetes mellitus: a major risk factor for cardiovascular disease. A joint editorial statement by the American Diabetes Association; The National Heart, Lung, and Blood Institute; The Juvenile Diabetes Foundation International; The National Institute of Diabetes and Digestive and Kidney Diseases; and The American Heart Association. *Circulation*.

[12] Diabetes UK, Diabetes in the UK 2010, Key statistics on diabetes (2010).

[13] Yellon DM, Hausenloy DJ (2007). Myocardial reperfusion injury. *The New England Journal of Medicine*.

[14] Haffner SM, Lehto S, Rönnemaa T, Pyörälä K, Laakso M (1998). Mortality from coronary heart disease in subjects with type 2 diabetes and in nondiabetic subjects with and without prior myocardial infarction. *The New England Journal of Medicine*.

[15] Malmberg K, Yusuf S, Gerstein HC (2000). Impact of diabetes on long-term prognosis in patients with unstable angina and non-Q-wave myocardial infarction: results of the OASIS (Organization to Assess Strategies for Ischemic Syndromes) registry. *Circulation*.

[16] Mathew V, Gersh BJ, Williams BA (2004). Outcomes in patients with diabetes mellitus undergoing percutaneous coronary intervention in the current era: a report from the prevention of REStenosis with tranilast and its outcomes (PRESTO) trial. *Circulation*.

[17] Alserius T, Hammar N, Nordqvist T, Ivert T (2006). Risk of death or acute myocardial infarction 10 years after coronary artery bypass surgery in relation to type of diabetes. *American Heart Journal*.

[18] Calafiore AM, Di Mauro M, Di Giammarco G (2003). Effect of diabetes on early and late survival after isolated first coronary bypass surgery in multivessel disease. *Journal of Thoracic and Cardiovascular Surgery*.

[19] Thourani VH, Weintraub WS, Stein B (1999). Influence of diabetes mellitus on early and late outcome after coronary artery bypass grafting. *Annals of Thoracic Surgery*.

[20] Paulson DJ (1997). The diabetic heart is more sensitive to ischemic injury. *Cardiovascular Research*.

[21] Yellon DM, Downey JM (2003). Preconditioning the myocardium: from cellular physiology to clinical cardiology. *Physiological Reviews*.

[22] Hausenloy DJ, Yellon DM (2008). Remote ischaemic preconditioning: underlying mechanisms and clinical application. *Cardiovascular Research*.

[23] Andreadou I, Iliodromitis EK, Koufaki M, Kremastinos DT (2008). Pharmacological pre- and post- conditioning agents: reperfusion-injury of the heart revisited. *Mini-Reviews in Medicinal Chemistry*.

[24] Murry CE, Jennings RB, Reimer KA (1986). Preconditioning with ischemia: a delay of lethal cell injury in ischemic myocardium. *Circulation*.

[25] Ferdinandy P, Schulz R, Baxter GF (2007). Interaction of cardiovascular risk factors with myocardial ischemia/reperfusion injury, preconditioning, and postconditioning. *Pharmacological Reviews*.

[26] Jones SP, Girod WG, Granger DN, Palazzo AJ, Lefer DJ (1999). Reperfusion injury is not affected by blockade of P-selectin in the diabetic mouse heart. *American Journal of Physiology*.

[27] Kersten JR, Toller WG, Gross ER, Pagel PS, Warltier DC (2000). Diabetes abolishes ischemic preconditioning: role of glucose, insulin, and osmolality. *American Journal of Physiology*.

[28] Lefer DJ, Scalia R, Jones SP (2001). HMG-CoA reductase inhibition protects the diabetic myocardium from ischemia-reperfusion injury. *The FASEB Journal*.

[29] Fiordaliso F, Leri A, Cesselli D (2001). Hyperglycemia activates p53 and p53-regulated genes leading to myocyte cell death. *Diabetes*.

[30] Marfella R, D’Amico M, Di Filippo C (2002). Myocardial infarction in diabetic rats: role of hyperglycaemia on infarct size and early expression of hypoxia-inducible factor 1. *Diabetologia*.

[31] Ebel D, Müllenheim J, Fräßdorf J (2003). Effect of acute hyperglycaemia and diabetes mellitus with and without short-term insulin treatment on myocardial ischaemic late preconditioning in the rabbit heart in vivo. *Pflugers Archiv*.

[32] Su H, Sun X, Ma H (2007). Acute hyperglycemia exacerbates myocardial ischemia/reperfusion injury and blunts cardioprotective effect of GIK. *American Journal of Physiology*.

[33] Desrois M, Clarke K, Lan C (2010). Upregulation of eNOS and unchanged energy metabolism in increased susceptibility of the aging type 2 diabetic GK rat heart to ischemic injury. *American Journal of Physiology*.

[34] Rees DA, Alcolado JC (2005). Animal models of diabetes mellitus. *Diabetic Medicine*.

[35] Chatzigeorgiou A, Halapas A, Kalafatakis K, Kamper EF (2009). The use of animal models in the study of diabetes mellitus. *In Vivo*.

[36] Yoon JW, Jun HS (2001). Cellular and molecular pathogenic mechanisms of insulin-dependent diabetes mellitus. *Annals of the New York Academy of Sciences*.

[37] Bolzán AD, Bianchi MS (2002). Genotoxicity of streptozotocin. *Mutation Research*.

[38] Like AA, Rossini AA (1976). Streptozotocin induced pancreatic insulitis: new model of diabetes mellitus. *Science*.

[39] Wilson GL, Leiter EH (1990). Streptozotocin interactions with pancreatic *β* cells and the induction of insulin-dependent diabetes. *Current Topics in Microbiology and Immunology*.

[40] Lenzen S (2008). The mechanisms of alloxan- and streptozotocin-induced diabetes. *Diabetologia*.

[41] Knowler WC, Barrett-Connor E, Fowler SE (2002). Reduction in the incidence of type 2 diabetes with lifestyle intervention or metformin. *The New England Journal of Medicine*.

[42] Portha B, Serradas P, Bailbe D, Suzuki KI, Goto Y, Giroix MH (1991). *β*-Cell insensitivity to glucose in the GK rat, A spontaneous nonobese model for type II diabetes. *Diabetes*.

[43] Howarth FC, Shafiullah M, Qureshi MA (2007). Chronic effects of type 2 diabetes mellitus on cardiac muscle contraction in the Goto-Kakizaki rat. *Experimental Physiology*.

[44] Kawano K, Hirashima T, Mori S, Saitoh Y, Kurosumi M, Natori T (1992). Spontaneous long-term hyperglycemic rat with diabetic complications: Otsuka Long-Evans Tokushima Fatty (OLETF) strain. *Diabetes*.

[45] Leonard BL, Watson RN, Loomes KM, Phillips ARJ, Cooper GJ (2005). Insulin resistance in the Zucker diabetic fatty rat: a metabolic characterisation of obese and lean phenotypes. *Acta Diabetologica*.

[46] Kobayashi K, Forte TM, Taniguchi S, Ishida BY, Oka K, Chan L (2000). The db/db mouse, a model for diabetic dyslipidemia: molecular characterization and effects of western diet feeding. *Metabolism: Clinical and Experimental*.

[47] Zhang B, Graziano MP, Doebber TW (1996). Down-regulation of the expression of the obese gene by an antidiabetic thiazolidinedione in Zucker diabetic fatty rats and db/db mice. *Journal of Biological Chemistry*.

[48] Hadour G, Ferrera R, Sebbag L, Forrat R, Delaye J, De Lorgeril M (1998). Improved myocardial tolerance to ischaemia in the diabetic rabbit. *Journal of Molecular and Cellular Cardiology*.

[49] Schaffer SW, Croft CB, Solodushko V (2000). Cardioprotective effect of chronic hyperglycemia: effect on hypoxia- induced apoptosis and necrosis. *American Journal of Physiology*.

[50] Oliveira PJ, Rolo AP, Seiãça R, Palmeira CM, Santos MS, Moreno AJM (2001). Decreased susceptibility of heart mitochondria from diabetic GK rats to mitochondrial permeability transition induced by calcium phosphate. *Bioscience Reports*.

[51] Nawata T, Takahashi N, Ooie T, Kaneda K, Saikawa T, Sakata T (2002). Cardioprotection by streptozotocin-induced diabetes and insulin against ischemia/reperfusion injury in rats. *Journal of Cardiovascular Pharmacology*.

[52] Ooie T, Takahashi N, Nawata T (2003). Ischemia-induced translocation of protein kinase C-*ε* mediates cardioprotection in the streptozotocin-induced diabetic rat. *Circulation Journal*.

[53] Ravingerová T, Neckář J, Kolář F (2003). Ischemic tolerance of rat hearts in acute and chronic phases of experimental diabetes. *Molecular and Cellular Biochemistry*.

[54] Kristiansen SB, Løfgren B, Støttrup NB (2004). Ischaemic preconditioning does not protect the heart in obese and lean animal models of type 2 diabetes. *Diabetologia*.

[55] Tsang A, Hausenloy DJ, Mocanu MM, Carr RD, Yellon DM (2005). Preconditioning the diabetic heart: the importance of Akt phosphorylation. *Diabetes*.

[56] Ma G, Al-Shabrawey M, Johnson JA (2006). Protection against myocardial ischemia/reperfusion injury by short-term diabetes: enhancement of VEGF formation, capillary density, and activation of cell survival signaling. *Naunyn-Schmiedeberg’s Archives of Pharmacology*.

[57] Chu LM, Osipov RM, Robich MP (2010). Is hyperglycemia bad for the heart during acute ischemia?. *Journal of Thoracic and Cardiovascular Surgery*.

[58] Shi-ting W, Mang-hua X, Wen-ting C, Feng-hou G, Zhu-ying G Study on tolerance to ischemia-reperfusion injury and protection of ischemic preconditioning of type1 diabetes rat heart.

[59] Mehta JL, Rasouli N, Sinha AK, Molavi B (2006). Oxidative stress in diabetes: a mechanistic overview of its effects on atherogenesis and myocardial dysfunction. *International Journal of Biochemistry and Cell Biology*.

[60] Boudina S, Abel ED (2007). Diabetic cardiomyopathy revisited. *Circulation*.

[61] Jonas M, Reicher-Reiss H, Boyko V, Behar S, Grossman E (2003). Hospital and 1-year outcome after acute myocardial infarction in patients with diabetes mellitus and hypertension. *Journal of Human Hypertension*.

[62] Tanaka K, Kehl F, Gu W (2002). Isoflurane-induced preconditioning is attenuated by diabetes. *American Journal of Physiology*.

[63] Desrois M, Sidell RJ, Gauguier D, Davey CL, Radda GK, Clarke K (2004). Gender differences in hypertrophy, insulin resistance and ischemic injury in the aging type 2 diabetic rat heart. *Journal of Molecular and Cellular Cardiology*.

[64] Bulhak AA, Jung C, Östenson CG, Lundberg JO, Sjoquist PO, Pernow J (2009). PPAR-*α* activation protects the type 2 diabetic myocardium against ischemia-reperfusion injury: involvement of the PI3-kinase/Akt and NO pathway. *American Journal of Physiology*.

[65] Matsumoto S, Cho S, Tosaka S (2009). Pharmacological preconditioning in type 2 diabetic rat hearts: the roles of mitochondrial atp-sensitive potassium channels and the phosphatidylinositol 3-kinase-akt pathway. *Cardiovascular Drugs and Therapy*.

[66] Thangarajah H, Yao D, Chang EI (2009). The molecular basis for impaired hypoxia-induced VEGF expression in diabetic tissues. *Proceedings of the National Academy of Sciences of the United States of America*.

[67] Murphy E, Steenbergen C (2007). Preconditioning: the mitochondrial connection. *Annual Review of Physiology*.

[68] Hausenloy DJ, Yellon DM (2011). The therapeutic potential of ischemic conditioning: an update. *Nature Reviews Cardiology*.

[69] Ovize M, Baxter GF, Di Lisa F (2010). Postconditioning and protection from reperfusion injury: where do we stand: position Paper from the Working Group of Cellular Biology of the Heart of the European Society of Cardiology. *Cardiovascular Research*.

[70] Bouchard JF, Lamontagne D (1998). Protection afforded by preconditioning to the diabetic heart against ischaemic injury. *Cardiovascular Research*.

[71] De Jong JW, De Jonge R, Keijzer E, Bradamante S (2000). The role of adenosine in preconditioning. *Pharmacology and Therapeutics*.

[72] O’Rourke B (2004). Evidence for mitochondrial K+ channels and their role in cardioprotection. *Circulation Research*.

[73] McIntosh VJ, Lasley RD (2012). Adenosine receptor-mediated cardioprotection: are all 4 subtypes required or redundant?. *Journal of Cardiovascular Pharmacology and Therapeutics*.

[74] Kamata K, Miyata N, Kasuya Y (1989). Functional changes in potassium channels in aortas from rats with streptozotocin-induced diabetes. *European Journal of Pharmacology*.

[75] Hausenloy DJ, Maddock HL, Baxter GF, Yellon DM (2002). Inhibiting mitochondrial permeability transition pore opening: a new paradigm for myocardial preconditioning?. *Cardiovascular Research*.

[76] Kersten JR, Montgomery MW, Ghassemi T (2001). Diabetes and hyperglycemia impair activation of mitochondrial KATP channels. *American Journal of Physiology*.

[77] Hausenloy DJ, Tsang A, Yellon DM (2005). The reperfusion injury salvage kinase pathway: a common target for both ischemic preconditioning and postconditioning. *Trends in Cardiovascular Medicine*.

[78] Hotta H, Miura T, Miki T (2010). Short communication: angiotensin II type 1 receptor-mediated upregulation of calcineurin activity underlies impairment of cardioprotective signaling in diabetic hearts. *Circulation Research*.

[79] Gross ER, Hsu AK, Gross GJ (2006). The JAK/STAT pathway is essential for opioid-induced cardioprotection: JAK2 as a mediator of STAT3, Akt, and GSK-3*β*. *American Journal of Physiology*.

[80] Baker JE (2005). Erythropoietin mimics ischemic preconditioning. *Vascular Pharmacology*.

[81] Gooch JL, Barnes JL, Garcia S, Abboud HE (2003). Calcineurin is activated in diabetes and is required for glomerular hypertrophy and ECM accumulation. *American Journal of Physiology*.

[82] Huisamen B, Pêrel SJC, Friedrich SO, Salie R, Strijdom H, Lochner A (2011). ANG II type i receptor antagonism improved nitric oxide production and enhanced eNOS and PKB/Akt expression in hearts from a rat model of insulin resistance. *Molecular and Cellular Biochemistry*.

[83] Ghaboura N, Tamareille S, Ducluzeau PH (2011). Diabetes mellitus abrogates erythropoietin-induced cardioprotection against ischemic-reperfusion injury by alteration of the RISK/GSK-3*β* signaling. *Basic Research in Cardiology*.

[84] Gross ER, Hsu AK, Gross GJ (2007). Diabetes abolishes morphine-induced cardioprotection via multiple pathways upstream of glycogen synthase kinase-3*β*. *Diabetes*.

[85] Miki T, Miura T, Hotta H (2009). Endoplasmic reticulum stress in diabetic hearts abolishes erythropoietin-induced myocardial protection by impairment of phospho-glycogen synthase kinase-3*β*-mediated suppression of mitochondrial permeability transition. *Diabetes*.

[86] Miura T, Nishihara M, Miki T (2009). Drug development targeting the glycogen synthase kinase-3*β* (GSK-3*β*)-mediated signal transduction pathway: role of GSK-3*β* in myocardial protection against ischemia /reperfusion injury. *Journal of Pharmacological Sciences*.

[87] Cheng H, Woodgett J, Maamari M, Force T (2011). Targeting GSK-3 family members in the heart: a very sharp double-edged sword. *Journal of Molecular and Cellular Cardiology*.

[88] Sivaraman V, Hausenloy DJ, Wynne AM, Yellon DM (2010). Preconditioning the diabetic human myocardium. *Journal of Cellular and Molecular Medicine*.

[89] Ravingerova T, Adameova A, Matejikova J (2010). Subcellular mechanisms of adaptation in the diabetic myocardium: relevance to ischemic preconditioning in the nondiseased heart. *Experimental and Clinical Cardiology*.

[90] Walker DM, Walker JM, Pugsley WB, Pattison CW, Yellon DM (1995). Preconditioning in isolated superfused human muscle. *Journal of Molecular and Cellular Cardiology*.

[91] Speechly-Dick ME, Graver GJ, Yellon DM (1995). Does ischemic preconditioning in the human involve protein kinase C and the ATP-dependent K+ channel? Studies of contractile function after simulated ischemia in an atrial in vitro model. *Circulation Research*.

[92] Kloner RA, Rezkalla SH (2006). Preconditioning, postconditioning and their application to clinical cardiology. *Cardiovascular Research*.

[93] Ghosh S, Standen NB, Galiñanes M (2001). Failure to precondition pathological human myocardium. *Journal of the American College of Cardiology*.

[94] Smith JM, Wahler GM (1996). ATP-sensitive potassium channels are altered in ventricular myocytes from diabetic rats. *Molecular and Cellular Biochemistry*.

[95] Hassouna A, Loubani M, Matata BM, Fowler A, Standen NB, Galiñanes M (2006). Mitochondrial dysfunction as the cause of the failure to precondition the diabetic human myocardium. *Cardiovascular Research*.

[96] Linares-Palomino J, Husainy MA, Lai VK, Dickenson JM, Galiñanes M (2010). Selective blockade of protein kinase B protects the rat and human myocardium against ischaemic injury. *Journal of Physiology*.

[97] Hausenloy DJ, Mwamure PK, Venugopal V (2007). Effect of remote ischaemic preconditioning on myocardial injury in patients undergoing coronary artery bypass graft surgery: a randomised controlled trial. *The Lancet*.

